# Using the Paleobiology Database to Teach Scientific Practices: an Approach Aligned to Relational Culture Theory

**DOI:** 10.1093/iob/obag028

**Published:** 2026-06-12

**Authors:** Rebecca M Price

**Affiliations:** School of Interdisciplinary Arts and Sciences, University of Washington Bothell, Box 358530, 17927 113th Ave N Bothell, WA, USA

## Abstract

This teaching innovations article explains how to develop a course-based undergraduate research experience built upon data from the Paleobiology Database with the principles of relational culture theory. The approach integrates psychosocial skills associated with research, like persistence through frustration, with scientific skills, such as observation, statistical analysis, locating and reading primary literature, and writing a scientific paper. Specifically, this course was designed to align with The Five Good Things in relational culture theory: to develop positive learning ethos, to create knowledge, development of self-worth, action or movement toward deeper learning, and a desire for continued learning. To achieve these aspects of relational culture theory, the course uses a survey—issued on the first day of class and revisited throughout the term—to establish a connection between the instructor and students; weekly observations of photos of fossils to make data from the Paleobiology Database less abstract; and an alternative grading scheme that emphasizes spiral feedback. This course meets once a week, with extra homework in lieu of additional class time. The first part of the course teaches paleobiology content through interactive lectures, carefully selected readings, and short videos. Students use collaborative note-taking software to discuss readings and videos that inspire research topics. The second part of the course is about analyzing paleobiological data, and students learn different statistical tests they can apply; simultaneously, they formulate their research hypotheses and download their data. This section of the course concludes with students analyzing their own data. In the third part of the course, students use primary literature to both justify the study and interpret the results they obtained. The final project is a scientific paper, which some students choose to submit to the campus research journal. Relational culture theory adds value to the research experience, and the strategies that build it into this course can be adopted to other contexts. In the future, focus group interviews with undergraduates who have taken the course could offer a nuanced analysis of the simultaneous effects of the approaches.

## Resumen

Este artículo sobre innovaciones pedagógicas explica cómo desarrollar cursos de experiencias investigativas para estudiantes universitarios basada en la Base de Datos de Paleobiología con los principios de la teoría de la cultura relacional. Este método integra habilidades psicosociales asociadas a la investigación (como la perseverancia ante la frustración) con habilidades científicas (como la observación, el análisis estadístico, la búsqueda y lectura de literatura primaria y la redacción de un artículo científico). Específicamente, este curso se diseñó para alinearse con los Cinco Elementos Clave de la teoría de la cultura relacional: desarrollar una ética de aprendizaje positiva, generar conocimiento, fomentar la autoestima, impulsar un aprendizaje más profundo, y generar el deseo de seguir aprendiendo. Para lograr estos aspectos de la teoría de la cultura relacional, el curso utiliza una encuesta—que se realiza el primer día de clase y se revisa a lo largo del trimestre—para establecer una conexión entre los profesores y los estudiantes; observaciones semanales de fotografías de fósiles para que los datos de la Base de Datos de Paleobiología sean menos abstractos; y un sistema de evaluación alternativo que enfatiza comentarios en espiral. Este curso se imparte una vez por semana, con tareas adicionales en lugar de tiempo extra de clase. La primera parte del curso enseña paleobiología mediante de clases interactivas, lecturas cuidadosamente seleccionadas y vídeos cortos. Los estudiantes utilizan software colaborativo para tomar notas y debatir sobre las lecturas y los vídeos que les inspiran temas de investigación. La segunda parte se centra en el análisis de datos paleobiológicos, donde los estudiantes aprenden diferentes pruebas estadísticas que pueden aplicar; simultáneamente, formulan sus hipótesis de investigación y descargan sus datos. Esta sección concluye con el análisis de los propios datos por parte de los estudiantes. En la tercera parte, utilizan literatura primaria para justificar el estudio e interpretar los resultados obtenidos. El proyecto final consiste de escribir un artículo científico, que algunos estudiantes optan enviar a la revista de investigación del campus. La teoría de la cultura relacional enriquece la experiencia de investigación, y las estrategias que la integran en este curso pueden adaptarse a otros contextos. En el futuro, entrevistar a grupos focales de estudiantes universitarios que hayan cursado la asignatura podría ofrecer un análisis más detallado de los efectos simultáneos de los diferentes enfoques.

## Introduction

At a time when scientific thinking is often dismissed, it is essential for undergraduates to become well-versed in understanding how scientific knowledge is produced and even to be involved in creating that knowledge through inquiry and experimentation. When students participate in course-based undergraduate research experiences (CUREs), they gain these insights. CUREs benefit all students, not just those going on to graduate school in our areas of expertise (Bangera and Brownell 2014).

CUREs can offer experiences similar to those gained from independent research experience in research labs, with hands-on mentoring when certain scientific problems arise. However, CUREs are more scalable, because they become a part of the curriculum, instructing all students in a course (Auchincloss et al. 2014; Bangera and Brownell 2014). Rather than the privileged few who know the advantages of pursuing research experience in a lab, and the even more select few that can afford to volunteer in a lab rather than working for pay, CUREs are an accessible opportunity open to all.

Other authors have already published in *Integrative Organismal Biology* about the benefits of using CUREs to teach concepts and skills necessary to conduct research in organismal biology (e.g., Daniels et al. 2026; Oufiero 2019; Price et al. 2020). These articles reference a paper that defines the attributes of a CURE (Auchincloss et al. 2014): that the students use scientific practices, are involved in discovery, engage in relevant research, collaborate, and iterate on previous research. Together, engaging in these practices on a topic that students choose, increases ownership over course activities, leading to deeper learning that students find more meaningful (Corwin et al. 2018; National Academies of Sciences, Engineering, and Medicine 2025).

This paper adds two components to the existing literature about CUREs based in organismal biology. First, it outlines a CURE that relies on data from the Paleobiology Database (https://paleobiodb.org/). Given that I teach at a university with 10 -week terms, developing a CURE around data that are already collected allows me to focus on other aspects of scientific thinking. Second, I draw on relational culture theory to explain structures built into the course that help students deal with the emotional upheaval so common in research (Schwarz 2019).

### Overview of a paleobiology CURE

I developed a CURE based on the Paleobiology Database (https://paleobiodb.org/). The learning goals of this course, *Science Methods and Practice*, are to

1. conduct scientific research and assume a scientific identity.
2. use statistics to analyze quantitative data, and present the results of the analyses in graphs and in words.
3. locate, read, and understand primary literature about a research topic.
4. adopt a collaborative approach to doing science, including conducting peer review by working with classmates to exchange ideas, share resources, and review each other’s work.
5. write a scientific paper that presents the results of original, scientific research and that places that work in the context of primary literature from that discipline.

The course meets requirements for majors in conservation and restoration ecology; data visualization; earth systems science; environmental studies; and science, technology and society. It also qualifies as credits for science distribution, quantitative thinking, and writing. It is highly unusual for me to have students with a background in geology, and none have had a background in paleobiology.

The Paleobiology Database makes the data accessible to newcomers. The navigator on the site is an interactive map that shows the distributions of fossils through time on either a map with the current configuration of the continents or with configurations at different times through geologic history.

### Relational culture theory

The psychosocial literature on effective CUREs focuses not just on the scientific skills that are gained, but also the affective growth that students experience (Auchincloss et al. 2014). Students grow in confidence, self-efficacy, and mindset. They normalize asking questions and working collaboratively with peers and with their instructors. Research can be incredibly frustrating to conduct, and thus many of us—faculty and students alike—need emotional support while we do it (Schwarz 2019; Cooper et al. 2020).

Persisting through frustrating learning experiences is often associated with learning from instructors attuned to an ethics of care (Noddings 1988; Schwarz 2019) who emphasize the joy in curiosity (Shay 2025). Ethics of care is an educational theory developed by Nell Noddings in the 1980s (Noddings 1988). Built for a K12 audience, the ethics of care emphasizes the relationship between instructor and student that can also be adapted to university students (Shukla et al. 2022). Strong faculty-student relationships are critical for retaining a diversity of students by increasing a sense of belonging (National Academies of Sciences, Engineering, and Medicine 2025; Simpfenderfer and Mitic 2025). Noddings concluded that these relationships are built on challenges that encourage growth, rather than niceness and easy-ness. It is also worth noting that persistence and support are not sufficient for excellence; for students with circumstantial stresses, including food, housing, and financial insecurity, care giving responsibilities, and threats to personal safety, no amount of care from a single instructor can balance the weight of systemic inequities (Gorski 2016; Harper 2010).

Noddings’s foundational work (Noddings 2003), in conjunction with other scholars like bell hooks (hooks 1994), have combined to inspire relational culture theory (RCT) and its application to education (Schwarz 2019). RCT offers guidance on how to generate meaningful interactions. Schwartz (2019) helps instructors enact RCT by presenting The Five Good Things that enhance work relationships with students: energy, knowledge, sense of worth, action or movement, and desire for more connection. While the Five Good Things can build relationships among students in a class, I focus here on the work relationships between instructor and students. I also elaborate on the significance of each Good Thing with respect to how they are practiced in CUREs.

When an instructor and student are engaged in conversation in ways that deepen both of their understandings about a scientific conundrum, there’s an energy and joy associated with the conversation. This is the positive **energy** that RCT values (Schwarz 2019). Such conversations are typical in CUREs, and even students who were once reticent to talk to their professor engage in these dialogs—an attribute that increases students’ inclusion in science (Bangera and Brownell 2014).

Interactions between people can also lead to **knowledge**. Any type of assessment—including in-class activities and homework—are ways to increase knowledge (Schwarz 2019). In a CURE, learning is open-ended enough that discoveries are being made (Auchincloss et al. 2014). Even tasks as mundane as an email are creating knowledge (Schwarz 2019). These mundane tasks can inspire more curiosity, which leads to more knowledge.

Validating that knowledge creation and explaining how working on assignments contributes to growth are also important aspects of learning. Enacting them increases students’ sense of **self-worth** (Schwarz 2019). The questions in a CURE are novel and meaningful, justified by gaps in the primary literature (Auchincloss et al. 2014). By creating new knowledge, students develop pride and participate in intellectual conversations.

Research inherently includes frustrating aspects, so part of teaching a CURE is supporting students through that frustration, helping them develop the skills to persist and to troubleshoot (National Academies of Sciences, Engineering, and Medicine 2025). That moment of getting unstuck, working through problems, is the **action or movement** described in RCT (Schwarz 2019).

Energy, common knowledge, sense of worth, and action or movement all synthesize into the last of The Five Good Things: **desire for more connection.** This is the opportunity to be in an ongoing scholarly conversation, to see not just one study that can be completed, but a multitude of them that can make a career (Schwarz 2019). Students can gain this connection to a greater scientific process by studying the primary literature in ways common in CUREs (Hoskins et al. 2011).

### Purpose

The goal of this paper is to share how I prioritize the instructor-student relationship in ways aligned to RCT while teaching a CURE built around the Paleobiology Database. The Methods section describes interventions that draw on RCT, then describes how I teach the course. The Results section presents my own reflections about how implementing RCT enriches this CURE. The Discussion contextualizes those reflections and offers insight into how others could adopt this course or sections of it, then concludes with limitations.

## Methods

### Course context

This CURE is a five-credit course, a standard course credit for the University of Washington. It is taught through a hybrid modality, in which students come to class once a week for two hours, and the rest of the instruction is asynchronous. The hybrid format affords the instructor more time for one-on-one mentoring.

Although the course is upper division and designed for third year students, there are no prerequisites because of the philosophy in the School of Interdisciplinary Arts and Sciences that any student should be able to participate in advanced learning. Only some of the students have taken an introductory statistics course. I have been teaching this course for 16 years, and none of the students have had background in paleobiology. Students can come from all majors, but most are majoring in biology or data visualization. The course is capped at 24 students, and it counts as part of the writing credit required for all majors at the University of Washington.

The course is highly scaffolded. Assignments build towards writing a scientific paper, which is the final project. Students use feedback from assignments to complete future ones, modeling spiral assessment (Carless 2019). This structure solves a common problem in instruction, that students ignore instructor feedback; instead, students are required to address feedback to complete the next task.

Class meetings are taught as workshops with a brief introduction to content and then activities designed to help students complete the work required for the next week.

Students have the opportunity to submit their final papers to the campus student research journal, *The CROW (Campus Research & Observational Writings)* although only a small percentage of students choose to do so. Over time, however, the articles that have been published in the campus journal help inspire future students: the future students indicate that seeing these publications makes the work of identifying a research question in an entire new area and completing a research study, all within one 10-week term, feel more achievable.

### Components of RCT

I have developed activities that consciously incorporate elements of RCT to promote learning relationships in the course. Each of these is tied to three or four of the Five Good Things; collectively, these activities appear in the three sections of the course, guaranteeing that the Five Good Things are also incorporated into each section (Table 1).

**Table 1:** Activities aligned to the Five Good Things of relational culture theory. Each activity occurs throughout the course.

	The Five Good Things					
Activity aligned to RCT	Energy	Knowledge	Sense of Worth	Action or movement	Desire for more connection	Frequency
**Introductory survey**	✓		✓	✓		First week, midterm, last week
**Alternative grading**		✓	✓	✓	✓	All assignments throughout term
**The photo that starts class**	✓	✓		✓		Every class meeting

#### Introductory survey

On the first day of class, students complete a “getting to know you” survey (Table 2). Many of the questions are intended to establish work relationships, as recommended by RCT. In science classes that do not teach sociocultural content, it can be hard to know students, because personally significant topics and perspectives are not necessarily part of the curriculum. However, sharing some personal perspectives that lie within a professional realm can improve learning (Schwarz 2019). For example, fostering skills around persistence can improve learning (Canning et al. 2024; Mackiewicz et al. 2023). Consequently, questions such as “What will you tell yourself to remind you that you belong in this class and can do well, even in the face of those challenges?” (Table 2) are aimed at improving self-efficacy and promoting belonging, both senses of self that promote retention in science (Louten 2024; Mackiewicz et al. 2023; Rainey et al. 2018; Simpfenderfer and Mitic 2025). Students take courses for different reasons, so asking “What would success in this course look like to you?” (Table 2) emphasizes that students can choose their level of engagement (see more under Alternative Grading). Students find it easier to learn from people with whom they share commonalities (Wheeless et al. 2011), so I share some information about myself and then ask them what we have in common. We have shared interests such as a love of science, crocheting, listening to similar music, parenting, layoffs, dogs, martial arts, an affection for grilled cheese sandwiches, and more. The other questions in the survey expand on these ideas and help me tailor the course to student interests.

**Table 2:** Questions asked in the “getting to know you survey”.


What name do you like to use in class? All of you who registered belong in this class. All of you will also be challenged at some point during the course. What will you tell yourself to remind you that you belong in this class and can do well, even in the face of those challenges? What would success in this course look like to you? What do you and I have in common? What major do you expect to obtain? What’s your greatest achievement in college so far? What’s a problem you want to solve in the world? Why are you taking a hybrid class [that meets only once a week, with more asynchronous work instead of more class time]? What do you gain from participating in class with this format? What do you compromise? Which of your skills will you rely on to succeed in this course? How might you expand those skills? What obligations or responsibilities may be barriers to or interfere with your success in this course? Are you OK with being called on in class? You will always have the option to pass. What else would you like me to know about you?

I use survey answers to become familiar with the students, but also to direct them to helpful services that the university provides, such as counselling services, emergency grants, and the food pantry.

We revisit the survey twice more (Table 1). At midterm, it helps students to review how they persist through challenges and their goals in class. In addition to reading through their answers, they can elaborate on them—this is particularly helpful given that barriers to success can emerge during the term. We also revisit the survey during the last class meeting, which helps me assess affective learning in the course, data I can use to improve the experiences of future students. I can use the data to better understand barriers to success that have emerged—especially given that stress is higher in the second half of the term—and whether there are structures in class that I can implement to mitigate those barriers. I also note when and how definitions of success change, how class content helps them identify strategies for addressing the world-wide problems that concern them. I find it heartening to note the commonalities students recognize that I share with them, achieving a goal of humanizing scientists.

#### The photo that starts class

Because the course has so much new content, it has the potential to overwhelm. Most students have not thought about geologic time or biodiversity—two vast concepts. To help connect their large data sets to real organisms, we start class by studying a photograph of a fossil specimen. I dim the lights, project a photograph, and ask students to write down what they notice and what they wonder about the specimen (Stachowiak 2022). As they are thinking, I share what I know about the natural history of the specimen. For example, while displaying a picture of a pyritized ammonite, I might talk about the process of pyritization, ammonite suture shapes, and how ammonites regulated their position in the water column. We might also talk about the Cretaceous/Paleogene extinction, and the new discovery of ammonites in the Paleogene (Machalski et al. 2025).

To include as many students as possible in this discussion, as well as the other activities, I use the practice of equitable call (Waugh and Andrews 2020). More commonly referred to as random call, this is the only method known to eliminate bias in who speaks in class. A number of strategies can make this process less intimidating for students (Waugh and Andrews 2020). For example, I find out who does not want to speak in whole class discussions (Table 2)—I want to avoid anxiety severe enough to interfere with learning (Cooper et al. 2021). Asking open-ended questions that have no obvious right or wrong answer helps folks be more willing to share. Encouraging students to write their thoughts before whole class discussion helps them organize what they would say, and it gives them something to read which can help them respond aloud. Finally, when I call on a student, I issue an invitation for them to contribute, e.g., “Would you like to share an observation you’ve made or a question you have?” I make sure students know that it is OK to refrain from answering. To orchestrate equitable call, I have a randomized list of student names that I bring with me to each class meeting. As students ask questions and share observations about photos like the ammonite, I answer as I can based on what I know, validating their observations and their ability to formulate questions.

#### Alternative grading

Alternative grading schemes that emphasize learning can increase sense of worth (Clark and Talbert 2023). I have implemented an alternative grading scheme that empowers student choice (Nilson 2014), promoting our relational connections (Schwarz 2019). The assignments are divided into those that are “required” and those that are “for excellence.” Students can complete the required material to earn a B. If they complete the “for excellence” assignments, perhaps because of a desire for more connection, then they can earn an A. The “required assignments” are all aligned to the task of writing a scientific paper about their own research question. Assignments categorized as “for excellence” introduce students to additional statistical analyses, more primary literature, and more aspects of the nature and process of science.

I grade assignments as complete or incomplete with a cut off at 80%. When I assign readings, I use shared annotation software, such as Perusall (https://www.perusall.com/) or Hypothesis (https://web.hypothes.is/), for students to take notes on readings collectively. I can discern quickly whether students have commented throughout an article and with sufficient depth, assessing how students practice communicating their new knowledge. The annotation software randomly arranges students into groups of eight; this group size keeps the amount of highlighting and number of comments about the reading at a balance between enough for engagement and too much to track. I answer many of the questions that students raise about the readings—but more important is the energy they build among each other through their dialogs.

Learning takes different people different amounts of time and effort, and it is based on the kind of formative response obtained through a feedback loop or, with multiple revisions, a feedback spiral (Carless 2019; Miller et al. 2025). Therefore, I accept resubmissions (Clark and Talbert 2023; Nilson 2014). When students receive an incomplete on an assignment that they attempted, they have one week in which to resubmit their work; this strategy helps them create movement or action through misunderstandings. They can only resubmit each assignment once, and they need to accompany it with a reflection (Table 3). Completing these questions encourages reflection and growth rather than grabbing for points. Limiting resubmissions to one opportunity per assignment helps students think deeply about their feedback (Clark and Talbert 2023).

**Table 3:** Questions that students answer when resubmitting work.


What did you do to improve your understanding before this revision? What resources did you use to study and prepare for this revision? What important mistakes were in the original, and how did you fix them?

Accepting—and even encouraging—resubmissions increases grading time for instructors, and I have developed some strategies that keep the workload manageable. First, assessing an assignment as incomplete or complete takes much less time than trying to discern the difference between percentage points (Nilson 2014). Second, I save detailed, individualized feedback for assignments related to writing the final paper. Doing so ensures that students will use the feedback that I provided. I carve out time in class for students to read the feedback to emphasize how important it is for their progress in the course and to afford the opportunity for them to ask questions about it. For lower stakes assignment, I provide feedback to the whole class, rather than to individuals. This approach means that it becomes the students’ job to discern what parts of the feedback apply to them, which increases their dexterity with self-evaluation. In sum, the benefits of resubmission far outweigh the additional grading time that the approach takes.

I post all assignments at the beginning of the term, giving each student a score of 0%. During the term, then, each assignment they complete increases their grades. I adopt this approach carefully, because it can be very disruptive to some students to see their grades start so low. Implementing it requires lots of discussion. For example, I emphasize the metaphor that students are climbing a mountain as they engage in the work of the course and see their grades increase. They reach the top of the mountain when they finish the course. This way, I explain, students are more keenly aware of their standing in the course as they move into the final project. They know the impact that completing the final project will have. The final paper requires that students revise extensively. So, it is possible for students to have completed all of their drafts to submit a final paper that is incomplete because it is not sufficiently revised. Thus, the approach of starting with a zero for the final grade avoids a false sense of security.

To help students understand their current grade, each week I show them the percentage they should have received if they completed the “required assignments” or both the “required” and “for excellence” assignments. I explain that they should be targeting their grade between these values. If students have been aiming to complete the “required assignments” but have not been able to do so, the “for excellence” assignments represent opportunities for them to increase their grade. To promote self-worth, I emphasize that students can complete any of the future assignments they choose, and that I am available to help them. I offer appointments with them as requested, in addition to office hours.

I have facilitated in-class conversations with students about moving to a different system—where future assignments are ungraded instead of assigned zeroes, but by midterm, they have adjusted to this approach and the metaphor of climbing the mountain. The votes have always led to keeping the system.

### Course format

Given that the University of Washington is on the quarter system, I have only 10-weeks in which to teach this course, with the final paper due in the eleventh week. Table 4 describes how I arrange the quarter, although instructors in the semester system can potentially take a more leisurely approach.

**Table 4:** Organization of the 10-week term.

Part I: Get Started	Weeks 1- 2
Part II: Analyze data	Weeks 3–4
Part III: Write the paper	Weeks 5–10

#### Part I: Get started

Students benefit from learning why studying the fossil record is so critically necessary. Paleobiology is intricately connected to understanding current ecosystems, and to illustrate the discipline’s impact, the course begins with two modules prepared by the Understanding Science team at the University of California Museum of Paleontology, University of California, Berkely.

First, in an interactive lecture, I discuss “Asteroids and Dinosaurs” (Understanding Science 2026), a lesson about the discovery of the asteroid impact triggering the Cretaceous-Paleogene extinction. The lesson demonstrates the unpredictability of the process of science, starting a shift in conceptualizing science as linear conceptualization (question-test-repeat) to a more realistic sense of its twists and turns (Price and Perez 2018). The content is ideal for this course, given enthusiasm for the topics of dinosaurs and their sudden extinction.

Second, we read in homework a review about latitudinal diversity gradients by the same team (Understanding Evolution 2026). The authors interspersed questions throughout the reading to help students check their own understanding. This reading introduces students to large scale, macroecological patterns. The lesson dips into the fossil record when considering some of Jablonski’s work looking at the tropics as either a cradle or museum of biodiversity (Jablonski et al. 2006). This assignment helps the students recognize the value of large databases in answering questions about macroecology and evolution over deep time scales.

More introductory assignments include watching short videos that introduce geologic time, taxonomy, the Paleobiology Database Navigator, the Paleocene-Eocene Thermal Maximum, and the large mammal extinctions in the Plio-Pleistocene of North and South America (Table 5). Like the readings, these videos are posted in shared annotation software, so that students can comment on them collaboratively. The videos introduce paleobiological topics that students can research in this class. I encourage students to build from one of three datasets I have downloaded from the PBDB: bivalves throughout the Phanerozoic, because this builds on my research background; mammals since their origin, because of the appeal of charismatic megafauna; and Paleocene/Eocene data for those interested in using the Paleocene-Eocene Thermal Maximum as a model for contemporary climate change.

**Table 5:** Videos that students watch for background information.


A Brief History of Geologic Time, https://youtu.be/rWp5ZpJAIAE (12 min) Your Place in the Family Tree, https://lms.hypothes.is/app/basic-lti-launch The Paleobiology Database Navigator, https://youtu.be/db2He3p-Jco (5 min) Science Bulletins: PETM—Unearthing Ancient Climate Change, https://youtu.be/db2He3p-Jco (8 min) In our Nature: The Mystery of Earth’s Disappearing Giants, https://youtu.be/95HP73Yemxo (21 minutes)

#### Part II: Analyze data

By the end of the “Get Started” section of the course, students have identified potential research areas that interest them. At this point, I meet with them individually to refine their interests into research hypotheses that are testable with data from the Paleobiology Database. These meetings are time consuming and form a bottleneck in the course.

Each meeting lasts about half an hour, and I record the meetings so that students have a record of what we discussed; this recording will become helpful later when they write the Materials and Methods and need to explain the decisions we made about what data to include in the analysis. Students usually decide to conduct their analysis at either the species or genus level. Data from the Paleobiology Database are stored at the specimen level, so summarizing information for a taxon must consolidate across several rows of specimen-level data. This consolidation can easily be done with pivot tables in Excel (Cohen 2015). Cleaning the data usually involves removing taxa not relevant to the students’ research question (for example, students only looking at plants in a dataset of all taxa alive in the Paleocene), specimens identified to a higher taxon than the students are using (e.g., specimens identified to family when students are working at the genus level), or specimens with missing data. Neither cleaning data nor conducting pivot tables are related to course outcomes, so I do both during our one-on-one meetings to set up the data for students to analyze. Students complete analyses in Microsoft Excel or Google Sheets.

The complexity of an analysis is constrained by the statistical tests that we learn in the course. All students learn a Mann Whitney U test—and to graph boxplots of data—to compare the medians of two groups. This test is useful when comparing extent or midpoints of latitudinal ranges of taxa across time periods.

Students completing “for excellence” assignments about statistics learn three additional tests: single factor ANOVA (with box-and-whisker plots), χ^2^ test (with bar charts), and linear regression (with scatter plots and a regression line). Students can use one of those tests to analyze their data. χ^2^ tests are particularly common for students comparing the relative proportions of two or more taxa in two different time periods.

Concurrently, students work with a librarian who introduces different scholarly databases for finding primary research articles related to their topic. One of the class meetings is devoted to finding scholarly literature and practicing statistics. Both the librarian and I attend, fielding questions as needed.

By the end of this section of the course, the students have a hypothesis, data, a completed statistical test, and at least three papers from the primary literature that they can use to justify why testing their hypothesis is important to the scholarly community.

#### Part III: Write the paper

After students have tested their hypotheses, they have the information they need to write the Materials and Methods and Results. A “for excellence” assignment asks students to evaluate a primary article to generate guidelines for how to write these sections of a scientific paper. I ask them to copy and paste the text of their article into a new document, and then to comment on specific aspects that model the writing style. This approach minimizes the use of generative artificial intelligence (AI) to complete the assignment, because it is more laborious to type several prompts into the AI than to describe one’s own observations. My larger goal, I explain, is for students to realize that they can turn any piece of writing into a template to mimic its style. I compile the students’ guidelines to write a template for the Materials and Methods and Results. A few weeks later, students complete a similar assignment to generate guidelines for writing the rest of a scientific paper (see a sample Paper Template in the Supplemental Materials).

After completing the Materials and Methods and Results sections, students take a quiz about how to organize their Introduction and Discussion. Grading the quiz is an efficient way for me to assess how students are justifying their research in the Introduction, and how they are composing rhetorical arguments to explain their results. Consequently, I am also assessing which students need to find more literature. Part of the quiz asks students to write what will become the last paragraph of the Introduction—a component of a paper that I often find to be the clearest statement of a paper’s purpose. To do so, they complete this template (following strategies in *They Say/I Say* (Graff et al. 2021)):

*In this paper, I test the hypothesis that $\rule{5pc}{0.5pt}$. The result that $\rule{3pc}{0.5pt}$ [brief description of the result]$\rule{2pc}{0.5pt}$ indicates that $\rule{3pc}{0.5pt}$ [brief summary of your claim]*.

When writing the Discussion, students struggle with explaining how papers interact with each other and with their own data. They need coaching to avoid a Discussion that reads as a series of paper summaries. Reading from *The Craft of Research* (Booth et al. 2016), students learn how to articulate claims and support those claims with reasons and evidence. I also provide a template that guides students to write dialogues among their sources:

*Give an example of how you are staging a dialogue among multiple sources in your Discussion. Complete this template to start getting your ideas in writing: Other researchers have found similar results. For example, $\rule{1.80pc}{0.5pt}$description of finding$\rule{3.4pc}{0.5pt}$ [citation]. As another example, $\rule{1.84pc}{0.5pt}$ description of finding$\rule{4.13pc}{0.5pt}$ [citation]. These authors interpret the results to mean that $\rule{4.85pc}{0.5pt}$ [citations.] On the other hand, some findings seem different. For example, $\rule{1.40pc}{0.5pt}$description of finding$\rule{3.05pc}{0.5pt}$ [citation], and $\rule{5.54pc}{0.5pt}$description of finding$\rule{1.51pc}{0.5pt}$ [citation]. To interpret these contradictory findings, I $\rule{7.08pc}{0.5pt}$.*

Because students’ own voices are critically important when writing, I next ask students to

*Rewrite your answer to the question above in your own voice; this exercise will move you away from the programmed, template language to something that is more genuinely you*.

After completing the quiz and receiving feedback about it, students are ready to write the whole paper.

Students have received comments from me on their Materials and Methods and Results, as well as on their Introduction and Discussion quiz. Therefore, I ask them to write a draft of a cover letter that responds to my feedback along with the whole paper. This helps them establish the practice of listing the requested changes and describing whether and how they have made them.

Throughout Part III: Write the Paper, students completing “for excellence” assignments learn about the peer review process, considering why peer review is important in science and how to write peer reviews (they read and comment on these papers: Voight and Hoogenboom (2012) and August and Brouwer (2024)). While only some students have read these articles, we still discuss their content as a whole class, preparing everyone to conduct peer reviews of each other’s work. Students review each other’s papers. As journals often do to solicit high quality peer reviews, I present the students with questions to answer (Table 6). The questions help them focus on substantive feedback rather than copy-editing; they are a combination of yes/no, comprehension, and open-ended responses that encourage evaluative reflection of the sort difficult for generative AI to do currently. I also grade the paper drafts, offering extensive feedback for students to revise and resubmit their work.

**Table 6:** Questions students answer during peer review of the paper. *questions that are especially difficult for generative AI to answer.


Does the Abstract briefly summarize each section of the paper? In your own words, what is the research hypothesis that your colleague is testing? In your own words, why is this research hypothesis important to test and relevant to the scientific community? * Are the methods appropriate for testing this hypothesis? Why? * Do the Results make sense to you? Why? * Can you determine the results from the Fig.(s) alone? In your own words, what is(are) the argument(s) your colleague makes in the Discussion? * How/why are the conclusions justified given the results and the research hypothesis? * What do you like best about this paper? * Is this paper complete? Why? * What else would you like to add?

Students need to revise their papers according to the feedback that their colleagues and I have provided. They also need to expand on their initial cover letters to incorporate the new suggestions. The final for the course consists of the revised paper and the cover letter. I also offer them a grade based on the quality of the improvement they made on their papers.

## Results

This Results section focuses on how implementing relational culture theory enriches a CURE. By focusing on RCT and The Five Good Things (energy, knowledge, sense of worth, action or movement, and desire for more connection, Schwarz 2019), I have realized that my goal is not to add elements that appeal to everyone in the course, but rather to make sure that all of the students enjoy a sample of the pedagogical strategies that I use to lift up their successes.

One measure of success for the course is that 11 students have published their papers in our campus research journal, *The CROW (Campus Research & Observational Writings)*. These papers make up 9% of the publications in the journal—a journal that publishes research from across disciplines—since 2018, the year of the first publication from the course. While I do not have much insight on why some students choose to submit, whether the course ends close to the deadline for submissions does seem to be a factor.

### Introductory survey

I use the survey to generate enthusiasm for the course content and tasks. As the term continues and students become more comfortable with me, they share more barriers. Common obstacles include aspects of neurodiversity, stresses to mood, financial insecurity, housing insecurity, and fears about United States Immigration and Customs Enforcement. This sharing allows me to point them to already existing campus resources and plan success for this course despite the barriers. I am clear about the kind of support I am not qualified to provide—for my own health and safety as well as the students’.

By validating students’ answers to the survey, and by sharing similar barriers, strategies for persistence that I and others have used, we create work relationships centered on persisting in the course. This persistence contributes to the success during Part I: Get Started, when students are dealing with a potentially overwhelming amount of new content; during Part II: Analyze Data for students who contend with math anxiety; and during Part III: Write the Paper during the challenging work of constructing arguments about their results with perspective from several primary articles.

Each section of the course has its own challenge, and persistence is required to navigate each. Persistence fosters a sense of worth. Moreover, each of these challenges is about creating knowledge, first with learning new material, and by the end of the course, with students’ own novel scientific discoveries. Building across parts of the course reflects the movement described in RCT. Every point during which students persist through a challenge, every time they get “unstuck” (Schwarz 2019, p. 18), they are engaging in more connection.

### The photo that starts class

Students make insightful observations about the photo that starts class. Their questions improve over the quarter, as does their willingness to ask those questions. Discussions lead to insights into the processes of fossilization, the discovery of fossils in the field, and the necessity of museum collections. Students also see that, despite my knowledge, there are some questions I cannot answer, either because I do not know or because the answers have yet to be discovered. I am modeling for them the directions I can take for my own deeper learning.

As with any class, some students arrive late—in this case, their lateness raises the possibility that some students do not feel that the photo that starts class is an important part of their learning.

The freestyling about the photo draws on The Five Good Things. It creates enthusiasm when students share what they have observed and ask questions, creating knowledge while practicing the fundamental scientific skill of observation. Sense of worth increases because I validate students’ observations and questions, using them to guide the natural history that I share. The action or movement this creates prepares them to engage more fully with their data.

### Alternative grading

Grades in the course have improved over time, with fewer students earning Cs and Ds than they did in earlier iterations. Some students indicate in their end of term evaluations that they appreciate that assignments are graded on complete/incomplete. Comments about “too much busy work” have almost vanished. Moreover, rather than agonizing over the differences in small percentages of a grade with large consequences for students (e.g., 69.4% instead of 70%), I spend my effort providing constructive criticism. For smaller assignments, I provide feedback to the whole class, so everyone benefits from it, and for students to learn how to discern which feedback is most appropriate for their own work. This focus on feedback aligns with The Five Good Things in RCT by sparking interest and encouraging learning.

The grading scheme is admittedly unusual, and it takes students time to get used to it. A few students do not like it, and the most objections are because grades start at 0%. However, I find the benefits outweigh the criticism. The approach plays into a metaphor of students climbing a mountain to finish the course—that they are meeting a challenge that builds from smaller bouts of effort throughout the term. The approach emphasizes the intellectual shift that students make, as described in RCT.

Assigning 0s to future assignments gives students a realistic sense of what they need to do to earn the grade they are aiming for, and a way to quantify their steady, cumulative successes over the term. Sometimes, students have a false sense of success when they have a high grade before a final is due; grades that are estimated only on past assignments can drop precipitously for students who do not do well on the final project. That drop damages students’ sense of worth. My expectation is that having a sense of what one has accomplished—and what one must still accomplish—helps students increase their sense of worth, in turn increasing a sense of belonging in and appreciation for scientific research.

The conversations generated through appointments, office hours, and feedback on assignments collectively contribute to a desire for more connection. Students are connecting themselves, through the paleobiological research community, to scientific endeavors more broadly.

After rounds of trial and error, I have found that tiering the course into “required” and “for excellence” assignments helps students prioritize tasks. The names matter. I used to call the “for excellence” assignments “optional,” but fewer students completed them when I used that nomenclature.

The students who appear to have the most difficult time with this grading scheme seem to be those who are capable of doing better work than they are used to doing, perhaps because they want to complete assignments instead of learn from them; this approach to completing assignments sometimes results in grades at or higher than the 80% threshold, but sometimes it does not. For students who have found that aiming for the middle has been a successful strategy for them to pass classes, my approach feels unjust. My strategy for dealing with that complaint is to focus on and celebrate the learning they are doing—in RCT terms, focusing on the knowledge they are acquiring and the discoveries they are making. For this population of students, I find it more challenging to engage them in action or the desire for more connection.

## Discussion

In this paper, I have demonstrated through reflection the way relational culture theory enhances a CURE taught with data from the Paleobiology Database. In this Discussion, I summarize the added value of teaching with a philosophy steeped in RCT, then discuss how transferable the RCT structures that I have adopted may be to other classes. I conclude by discussing the limitations of this CURE and future research that could study its efficacy more rigorously.

### Added value of RCT

It can be challenging to achieve the affective skills necessary to pursue scientific academic research (Cooper et al. 2020; Schwarz 2019; Shay 2025; Simpfenderfer and Mitic 2025), especially given that teaching them is often not part of science curricula. However, CUREs offer a chance to include them (Auchincloss et al. 2014; Bangera and Brownell 2014). Metacognition, for example, is an essential part of learning (National Academies of Sciences, Engineering, and Medicine 2018), but not explicitly taught in the curriculum the way it is, for example, in psychology, a field in which it is a core subject. Teaching with a philosophy rooted in RCT can be a way of adding content about psychosocial skills to CUREs.

Applying RCT to an alternative grading scheme, for example, puts into practice a fundamental aspect of teaching: feedback loops or, if feedback and improvement are ongoing, feedback spirals (e.g., Carless 2019). Instructors measure how students are doing, providing formative feedback for them to improve. Then, we reassess, and we give additional feedback. The alternative grading scheme I apply emphasizes that learning is cyclical. Students are indoctrinated into more traditional grading schemes that emphasize summative feedback; with summative feedback, they can fall prey to no longer engaging the material after an assignment is done (Carless 2019; Miller et al. 2025). Emotional support includes explaining why redoing an assignment is worthwhile, so I ask students what they have learned from the process of revision. I encourage students to explain and explore what they learn from revision.

RCT also adds value to learning by making it more enjoyable. Earlier in my teaching career, I quantified students learning gains through pre-post testing. I could demonstrate that my students were learning a great deal, although my end-of-term feedback often indicated that they were not necessarily enjoying my classes. Given the importance of scientific literacy, and the need for a public that values the process and results of scientific research, I have made a concerted effort over the last 15 years to have students learn as much as they had in the past, if not more, while enjoying the process. Students engage with, value, and enjoy the challenge and frustration of discovery.

### Transferability

I have explained a series of techniques that foster instructor-student relationships according to RCT. While I have tailored these techniques to my course environment, all of them can be used or adapted to many contexts. For example, an introductory survey can be administered in any class. A photo to start class can be adapted to whatever content is being taught—for example, biodiversity may be especially fun to share, as proposed by Davis-Berg (2009). A wealth of examples illustrate different grading schemes, including how they can be applied in exam-based science courses (Clark and Talbert 2023; Nilson 2014).

### Limitations and future work

As an instructor, the biggest challenge in teaching this course is helping students extract and clean their datasets. Most third-year college students do not know about pivot tables, for example, so they need help understanding how to rearrange data from the Paleobiology Database from the specimen level to species or genus-level analyses. Given that my students lack background in paleobiology, meeting with each student for about half an hour to refine the hypothesis and set up their data has been essential, even though it is time consuming.

Often CUREs are part of a professor’s research agenda, with results that are potentially publishable in scientific journals (Auchincloss et al. 2014). In this case, however, I no longer have an active research program in paleobiology. While the students are constrained to use data from the Paleobiology Database, they do not see their work through to journal publications. Instead, our campus research journal, *The CROW (Campus Research & Observational Writings)*, is a better choice for a publication venue. Of course, other instructors could have great success using the Paleobiology Database to inspire publications with undergraduate students either in classes or independent research experiences.

I have limited data about the impact of this approach. While a vast literature supports the techniques I have adopted, I have not formally studied the impact of my implementation of these techniques. Instead, this paper presents a case study on how to adopt RCT in a CURE. For other instructors looking to create CUREs, CUREnet (CUREnet n.d.) and the Council on Undergraduate Research (Council for Undergraduate Research 2018) are both excellent resources for finding CUREs that have been studied rigorously.

Future work could conduct focus group interviews with students in this or similar courses. Such data would offer a nuanced analysis of the simultaneous effects of the approaches I have adopted. Focus groups would be ideal for assessing the impact of the instructor-student relationship on learning to identify explicitly the impact of RCT in course design. Questions could target each of the Five Good Things, for example by asking “how does talking about a scientific discovery affect the way you feel about completing an assignment in this class?” to begin to assess positive energy. Existing surveys on science identity (e.g., Breitbeck et al. 2026) and belonging (e.g., Gopalan and Brady 2020) could supplement the results obtained from focus groups.

Given the small sample sizes within each course, the number of revisions that happen from year-to-year, the idiosyncrasies of each instructor implementing techniques that promote RCT, and rapid changes in the way science is politicized in the U.S., a quantitative study would be hard to interpret. However, quantitative tools are available, namely Lopatto’s Classroom Undergraduate Research Experience (CURE) survey that assesses students’ learning gains, shifts in attitudes towards science, and the quality of the students’ experience (Lopatto n.d.).The strategies I have described for this course, implement my best understanding of how people learn. Research on learning is always changing: our knowledge deepens and the way people learn changes over time. Thus, the content in this article represents a snapshot in time of how I teach this course. I will continue to update it.

## Supplementary Material

obag028_Supplemental_File

## References

[bib1] Auchincloss LC, Laursen SL, Branchaw JL, Eagan K, Graham M, Hanauer DI, Lawrie G, McLinn CM, Pelaez N, Rowland S, Towns M, Trautmann NM, Varma-Nelson P, Weston TJ, Dolan EL 2014. Assessment of course-based undergraduate research experiences: a meeting report. LSE 13:29–40. 10.1187/cbe.14-01-0004 PMC3940459 24591501

[bib2] August ET, Brouwer AF 2024. How to write an effective journal peer review using a staged writing approach: a best-practice guide for early-career researchers. Int J Epidemiol 53:dyae154. 10.1093/ije/dyae154 PMC11580681 39570678

[bib3] Bangera G, Brownell SE 2014. Course-based undergraduate research experiences can make scientific research more inclusive. LSE 13:602–6. 10.1187/cbe.14-06-0099 PMC4255347 25452483

[bib4] Booth WC, Colomb GC, Williams JM, Bizup J, FitzGerald WT 2016. The Craft of Research (4th ed.). Chicago, IL: University of Chicago Press.

[bib5] Breitbeck A, Ozyazici G, Tillotson JW, Wang Q 2026. The Student Science Identity (SSI) Questionnaire: a Multi-Group Validation Study Among US College Students. Journal of College Science Teaching 55:140–56. 10.1080/0047231X.2026.2628026

[bib6] Canning EA, White M, Davis WB 2024. Growth mindset messages from instructors improve academic performance among first-generation college students. LSE 23:ar14. 10.1187/cbe.23-07-0131 PMC11235104 38470818

[bib7] Carless D 2019. Feedback loops and the longer-term: towards feedback spirals. Assessment & Evaluation in Higher Education 44:705–14. 10.1080/02602938.2018.1531108

[bib8] Clark D, Talbert R 2023. Grading for Growth: A Guide to Alternative Grading Practices that Promote Authentic Learning and Student Engagement in Higher Education. New York, NY: Routledge.

[bib9] Cohen PA 2015. Using Lookup and Pivot Tables in Excel with the Paleobiology Database. Williamstown, MA: [YouTube] [cited 2026 Jun 26]. Available from: https://www.youtube.com/watch?v=mMrIHhtjGls

[bib10] Cooper KM, Gin LE, Barnes ME, Brownell SE 2020. An exploratory study of students with depression in undergraduate research experiences. LSE 19:ar19. 10.1187/cbe.19-11-0217 PMC8697659 32412838

[bib11] Cooper KM, Schinske JN, Tanner KD 2021. Reconsidering the share of a think–pair–share: emerging limitations, alternatives, and opportunities for research. LSE 20:fe1. 10.1187/cbe.20-08-0200 PMC8108495 33444105

[bib12] Corwin LA, Runyon CR, Ghanem E, Sandy M, Clark G, Palmer GC, Reichler S, Rodenbusch SE, Dolan EL 2018. Effects of discovery, iteration, and collaboration in laboratory courses on undergraduates’ research career intentions fully mediated by student ownership. LSE 17:ar20. 10.1187/cbe.17-07-0141 PMC5998318 29749845

[bib13] Council for Undergraduate Research . 2018. Council for Undergraduate Research. Gaithersburg, MD. https://www.cur.org/

[bib14] CUREnet . n.d. CUREnet. Retrieved May 28, 2021, from https://serc.carleton.edu/curenet/index.html

[bib15] Daniels J, McCallum M, Neal N, Nkwocha E, Machado SR, Carter H, Lam E, Peng C, O’Brien A, Wei N, Frederickson M, Tan J 2026. Introducing a novel course-based undergraduate research experience using duckweed as a model system. Integr Org Biol 8:obaf049. 10.1093/iob/obaf049 PMC12802901 41541504

[bib16] Davis-Berg EC 2009. The mystery organism of the day. The American Biology Teacher 71:139. 10.1662/005.071.0304

[bib17] Gopalan M, Brady ST 2020. College students’ sense of belonging: a national perspective. Educ Res 49:134–7. 10.3102/0013189X19897622 PMC11156233 38847009

[bib18] Gorski PC 2016. Poverty and the ideological imperative: a call to unhook from deficit and grit ideology and to strive for structural ideology in teacher education. Journal of Education for Teaching 42:378–86. 10.1080/02607476.2016.1215546

[bib19] Graff G, Birkenstein C, Durst R 2021. They Say/I Say (5th ed.). New York, NY: W. W. Norton & Company.

[bib20] Harper SR 2010. An anti-deficit achievement framework for research on students of color in STEM. In Harper S.R., Newman C.B. (Eds.), Students of color in STEM: New directions for institutional research (Vol. 148, pp. 63–74). San Francisco: Jossey-Bass.

[bib21] hooks b 1994. Teaching to Transgress: Education as the Practice of Freedom. New York, NY: Routledge.

[bib22] Hoskins SG, Lopatto D, Stevens LM 2011. The C.R.E.A.T.E. approach to primary literature shifts undergraduates’ self-assessed ability to read and analyze journal articles, attitudes about science, and epistemological beliefs. LSE 10:368–78. 10.1187/cbe.11-03-0027 PMC3228655 22135371

[bib23] Jablonski D, Roy K, Valentine JW 2006. Out of the tropics: evolutionary dynamics of the latitudinal diversity gradient. Science 314:102–6. 10.1126/science.1130880 17023653

[bib24] Lopatto D n.d.. CURE—Undergraduate Research Experience Surveys. Grinnell, IA. Retrieved April 27, 2026, from https://sure.sites.grinnell.edu/cure-survey/

[bib25] Louten J 2024. Fostering persistence in science, technology, engineering, and mathematics (STEM): creating an equitable environment that addresses the needs of undergraduate students. Journal of College Student Retention: Research, Theory & Practice 26:283–302. 10.1177/15210251211073574

[bib26] Machalski M, Olszewska-Nejbert D, Landman NH, Jagt JWM, Garb M, Milàn J 2025. Ammonite survival across the Cretaceous–Paleogene boundary confirmed by new data from Denmark. Sci Rep 15:45802. 10.1038/s41598-025-34479-1 PMC12756250 41476258

[bib27] Mackiewicz MR, Hosbein KN, Mason D, Ajjarapu R 2023. Integrating scientific growth and professional development skills in research environments to aid in the persistence of marginalized students. J. Chem. Educ. 100:199–208. 10.1021/acs.jchemed.2c00633 PMC10710303 38074344

[bib28] Miller S, Handelsman J, Knight JK, Knowles MA, Flowers S, Theisen C, Gormally C, Kennedy Z, Pfund C, Price RM, Sung R-J, Vemu S, Withers MD 2025. Scientific Teaching Course. Golden, CO: Codon Learning. https://tiny-earth.github.io/ScientificTeachingCourse/stable/

[bib29] National Academies of Sciences, Engineering, and Medicine . 2018. How People Learn II: Learners, Contexts, and Cultures. Washington, D.C.: The National Academies Press. 10.17226/24783

[bib30] National Academies of Sciences, Engineering, and Medicine . 2025. Transforming Undergraduate STEM Education: Supporting Equitable and Effective Teaching. Washington, D.C.: National Academies Press. 10.17226/2

[bib31] Nilson LB 2014. Specifications Grading: Restoring Rigor, Motivating Students, and Saving Faculty Time. New York, NY: Routledge.

[bib32] Noddings N 1988. An ethic of caring and its implications for instructional arrangements. American Journal of Education 96:215–30. 10.1086/443894

[bib33] Noddings N 2003. Caring: A feminine approach to ethics & moral education (2nd ed.). Berkeley, CA: University of California Press.

[bib34] Oufiero CE 2019. The organismal form and function lab-course: a new CURE for a lack of authentic research experiences in organismal biology. Integr Org Biol 1:obz021. 10.1093/iob/obz021 PMC7671133 33791536

[bib35] Price RM, Perez KE 2018. Many paths toward discovery: a module for teaching how science works. Journal of College Science Teaching 47:78–87. 10.2505/4/jcst18_047_03_78

[bib36] Price SA, Larouche O, Friedman ST, Corn KA, Wainwright PC, Martinez CM 2020. A CURE for a major challenge in phenomics: a practical guide to implementing a quantitative specimen-based undergraduate research experience. Integr Org Biol 2:obaa004. 10.1093/iob/obaa004 PMC7671122 33791548

[bib37] Rainey K, Dancy M, Mickelson R, Stearns E, Moller S 2018. Race and gender differences in how sense of belonging influences decisions to major in STEM. IJ STEM Ed 5:10. 10.1186/s40594-018-0115-6 PMC6310405 30631700

[bib38] Schwarz HL 2019. Connected Teaching: Relationship, Power, and Mattering in Higher Education. Sterling, VA: Stylus Publishing, LLC.

[bib39] Shay J 2025. Overcoming STEM Imposter Syndrome through Joyful Curiosity. In Camfield E.K., Joy-Centered Pedagogy in Higher Education (1st ed., pp.153–68). Oxford: Routledge. 10.4324/9781003532224-11

[bib40] Shukla SY, Theobald EJ, Abraham JK, Price RM 2022. Reframing educational outcomes: moving beyond achievement gaps. LSE 21:es2. 10.1187/cbe.21-05-0130 PMC9508913 35294255

[bib41] Simpfenderfer AD, Mitic R 2025. Belonging and balance: institutional, socioeconomic, and external demands’ impact on racial variation in time-to-degree among college completers. Res High Educ 67:3. 10.1007/s11162-025-09876-0

[bib42] Stachowiak B (Host). 2022. How to grade creative assignments [Audio podcast episode 430]. *Teaching in Higher Ed*. https://teachinginhighered.com/podcast/how-to-grade-creative-assignments/

[bib43] Understanding Evolution . 2026. Introducing the LDG. Berkeley, CA: University of California Museum of Paleontology. https://undsci.berkeley.edu/asteroids-and-dinosaurs-unexpected-twists-and-an-unfinished-story/

[bib44] Understanding Science . 2026. Asteroids and dinosaurs. Berkeley, CA: University of California Museum of Paleontology. https://undsci.berkeley.edu/asteroids-and-dinosaurs-unexpected-twists-and-an-unfinished-story/

[bib45] Voight ML, Hoogenboom BJ 2012. Publishing your work in a journal: understanding the peer review process. Int J Sports Phys Ther 7:452–60. PMC3474310 23091777

[bib46] Waugh AH, Andrews TC 2020. Diving into the details: constructing a framework of random call components. LSE 19:ar14. 10.1187/cbe.19-07-0130 PMC8697661 32357096

[bib47] Wheeless VE, Witt PL, Maresh M, Bryand MC, Schrodt P 2011. Instructor credibility as a mediator of instructor communication and students’ intent to persist in college. Communication Education 60:314–39. 10.1080/03634523.2011.555917

