# Supplementary material for: Using the Paleobiology Database to Teach Scientific Practices: an Approach Aligned to Relational Culture Theory
Source: Integr Org Biol. 2026 Jun 12;8(1):obag028. doi: 10.1093/iob/obag028 (PMC13750173; doi:10.1093/iob/obag028)
Supplement: Supplementary file 1 [file obag028_supplemental_file.docx]

# Supplemental Materials

## Template for the paper

[Please remove the material in square brackets [ ] from the template before submitting your paper.

A scientific paper follows what’s called the **IMRaD format**, named for the major sections of the paper: **Introduction, Materials and Methods, Results,** and **Discussion.** This document compiles the guidelines you’ve developed to write each section of a scientific paper.

Your audience for this paper is any scientist at UWB. In previous years, the best papers have been between 10 and 15 double-spaced pages long.

**Please do not use direct quotations in your work.** Instead, paraphrase what the author(s) says, making sure to include in-text citations. In some fields of study, direct quotations are common, but they are unusual in a scientific paper. Paraphrasing has the added benefit of giving you practice understanding different authors’ ideas.

Use CSE Year-Name style:

Sample in-text citations

1 author: (Thalib 2024)

2 authors: (Zhang & Bargicho 2023)

3 authors: (Chohan et al. 2023)

Use this template for writing your paper. Make sure to remove any text in [square brackets]. Note that everything is in one column (instead of two) and that the lines are numbered. This is what it looks like when we submit manuscripts to scientific journals. Everything is double spaced.

Here are some tips:

- Use the answers you wrote in the R.Intro&Discussion quiz. You basically outlined both the Intro and the Discussion in that assignment.
- You can use first person (I found that…) or third person (The results are…). It is common for paleobiologists to write in first person.
- How many primary references should you use? Well, that depends on how much info is in the references you’re reading. Most students have around 10.
- All your references should be scholarly references, such as primary literature and review papers.
- Try to use active voice.
- Be organized, clear, and concise.
- Only include information that is relevant to your subject.
- Plagiarism results in 0. ]

# [Title: state what you found, instead of what you’re studying. The title is clear and concise, uses keywords, and communicates the major impact of your study]

[your name]

[date of submission]

# Abstract

[Write this section last.

The abstract summarizes each of the sections you’ve already written. The first sentence sets the stage, explaining why the research captures the attention of a broad audience. Then, you move into the specific research hypothesis you’ve tested. Take one or two sentences to summarize the methods employed and a brief summary of your results. Conclude the abstract by explaining what your results mean, tying them to a broader context. Do all this in only 150 words! I recommend that your first draft is longer than this, and then that you edit it down to 150 words.

- 1-2 sentences on introduction (state the aim of the research by identifying the scientific problem and your particular research hypothesis)
- 1-2 sentences on materials and methods (what taxa did you study, and with what variables? what statistical test did you set up?)
- 1-2 sentences on the most important result
- 1-2 sentences on discussion (the major impact of the research)
- 150 words]

**Keywords:** [Include 5 to 10 keywords that convey the main topic and themes]

# Introduction

[Write at least 3 paragraphs to link your research question to the context provided by outside literature.

Paragraph 1: Hook the reader into the paper by beginning with the most interesting aspect of what you’re studying. Based on the primary literature you’ve read, what is the gap in knowledge that you’re trying to address? Be explicit with shortcomings or holes in arguments that have been made. This is where you bring in larger and bigger world significance and provide background information for context. Mention previous studies and what results were found from them/what was missing. Also use the primary literature to explain why your research topic is important and how it contributes to the scientific community and how it is beneficial. In the guidelines you wrote, many of you refer to this as the *justification for* or *significance of* the research.

Paragraph 2+: Narrow down to explain why your system—including species, geologic location, climate, geologic time, etc.—helps address that gap in knowledge. Tell the audience what is known about the topic to prepare them to understand your research. Include as much relevant background information as possible. You may need to include definitions, if you’re using words that are not common in this discipline. Everything known must be cited.

Last paragraph: Last paragraph starts “In this paper, I test the hypothesis…” After stating your research hypothesis, state your research prediction. This is the objective of the paper. It’s similar to, but distinct from, a thesis statement in other kinds of research papers. Remember that some readers will skip to the final paragraph of introduction in order to determine whether they should read your paper, so it is imperative to be explicit in what your study is testing.]

# Materials and Methods

The data [for all bivalves/mammals/all taxa alive in the Paleocene and Eocene] were downloaded from the Paleobiology Database on [date] (<https://paleobiodb>.org). From this spreadsheet, I extracted [these data] from this file.

[Clearly describe the criteria used to select your data. What data did we remove and why? This is where you can mention confounding factors and justify the choices you made about which data to include.]

I applied a [name of test] test to test the prediction that [state your prediction]. I chose a significance level (α) of [state your α] because [explain your reasoning].

[Describe the variables you used and how you defined them.]

All analyses were conducted in [Microsoft Excel, Google Spreadsheets, etc…].

# Results

**Figure 1.** [Graph title. Name of statistical test, p-value, other statistical results that you need to include.]

To test the hypothesis that [your hypothesis], I predicted that [your prediction]. I applied a [name of test] test to compare [variable 1] and [variable 2]. This test resulted in a p-value of [your p-value]. This p-value means there is a [fill this in] % chance that the relationships tested are a result of random processes. Therefore, I [say accept/fail to reject/reject] my statistical null hypothesis and [say either: accept/fail to reject/reject] my research prediction (Figure 1).

# Discussion

[The purpose of the Discussion is to interpret your Results in the context of past work. Start with a brief summary of the results and an overview of how you’ve organized your discussion. State whether the results confirmed or are inconsistent with your hypothesis. Keep this brief. This paragraph comes before a series of subsections, each with a label that says something about argument in that subsection. Good examples of subsection titles are: *Impact of megafauna extinctions on modern the distribution of mammals, Effect of human hunting. The Role of the Latitudinal Diversity Gradient in Preserving Species*.

Use present tense for in the Discussion, for example, these results mean that…

This section requires many relevant articles, and should be organized into different, clearly-labeled subsections. Using multiple subsections makes it easier for the reader to navigate.

- Delve into what the results mean. Explain them. Analyze and interpret the patterns and relationships presented in the Results section.
- Tie your results back to your research hypothesis with primary sources to explain your findings.
- Compare evidence from scholarly published work to your own, referring to your data when making key points. When you refer to scientific articles, you can even have row of citations in one sentence.
- Be upfront about uncertainties established credibility. Acknowledge weaknesses in your argument and respond to them.
- Explain why this result or study is important. How do these findings add to the scientific community? At the same time, what questions still need to be answered?
- By convention, the last subsection should be called Limitations and Future work.]

## [First subsection title; title communicates the theme of this claim]

[Within each subsection, begin each paragraph with a claim and argue that claim by integrating your results and several articles into each paragraph. You want the ideas of one source to play off of the ideas from another. Indicate how generalizable you think your results are for other study populations. ]

[several paragraphs using the primary literature to interpret your results and develop this claim]

## [Second subsection title; title communicates the theme of this claim]

[several paragraphs using the primary literature to interpret your results and develop this claim]

## [Continuing with section titles as necessary…]

## Limitations and future work

[Use citations in this section, too.]

# Acknowledgments

[Thank any colleagues that have helped you throughout the process of analyzing your data and writing your paper. This can include the [redacted], your classmates, including the folks who conducted your peer reviews. You may (but are certainly not required to) acknowledge your professor.]

# Literature Cited

[Only include references that you cited in-text. All of the papers that you cited in-text need to be presented in the References. This means that you’ve read a number of papers that don’t end up being mentioned at all in your final paper! Do not include the in-text personal communications in the References, but do include papers that are in review.

- Format with CSE guidelines, https://www.scientificstyleandformat.org/Tools/SSF-Citation-Quick-Guide.html, Year-Name style; for papers, use the Journal Article—print only format.

Sample in-text citations

1 author: (Nguyen 2022)

2 authors: (Greenwood & Earnshaw 2021)

3 authors: (Wood et al. 2023)

Sample reference:

Bechtold JS, Edwards RT, Naiman RJ. 2003. Biotic versus hydrologic control over seasonal nitrate leaching in a floodplain forest. Biogeochemistry. 63(1): 52-72.

- Only include references that you cited in-text.
- Use only scientifically credible sources.
- Alphabetize the references in this section.]

# Supplemental Materials

[These are the raw data you used in your analysis. You can include a table here or, if it’s a lot of data, upload a different file as a spreadsheet. This should not show your analysis—just the data you used to set up your analysis.]
